# Troubleshooting Vaginal Stents: A Critical Review of Common Problems and Solutions

**DOI:** 10.7759/cureus.66388

**Published:** 2024-08-07

**Authors:** Shruti Deshmukh, Madhu Priya, Sweta G Pisulkar, Surekha A Dubey, Arushi Beri, Akansha Bansod, Ritul Jain

**Affiliations:** 1 Prosthodontics, Crown and Bridge, Sharad Pawar Dental College and Hospital, Datta Meghe Institute of Higher Education and Research, Wardha, IND; 2 Prosthodontics, Sharad Pawar Dental College and Hospital, Datta Meghe Institute of Higher Education and Research, Wardha, IND

**Keywords:** patient compliance, silicone stent, mrkh syndrome, vaginal stenosis, vaginal stents

## Abstract

This review aims to examine the use of vaginal stents in clinical practice, specifically for treating vaginal agenesis and related complications and problem associated with vaginal stents. Vaginal agenesis, also known as Mayer-Rokitansky-Küster-Hauser (MRKH) syndrome, is a congenital disorder characterized by the uterus not developing or developing only partially. Vaginal stents are medical devices that preserve the structural integrity and patency of the vaginal canal after trauma or surgery. They play an important part in gynecological treatments such as post-radiation therapy, reconstructive surgery, and vaginal stenosis management. The review also discusses the primary applications of vaginal stents, such as preventing adhesions, healing mucosa, and maintaining patency. It also investigates frequent concerns associated with stent use, such as complications and the need for better designs. Vaginal stents are essential in a variety of therapeutic settings, providing major benefits in maintaining vaginal tissue and function. However, their use is fraught with complications, including the risk of infection, discomfort, and the possibility of inappropriate placement.

## Introduction and background

Vaginal agenesis refers to a condition where the uterus may only develop partially or not at all, more commonly known as Mayer-Rokitansky-Küster-Hauser (MRKH) syndrome [1]. There are major concerns regarding women’s health, including violence, breast and cervical cancer, and reproductive health problems, among which reproductive health is the most neglected; hence, we must re-commit to address them. Vaginal agenesis is estimated to occur in approximately one in every 4500 females [2]. A vaginal stent is designed to support and maintain the shape of the vagina. It is typically used in cases with surgical procedure or trauma that requires support for the vaginal walls. The McIndoe technique is used in vaginal reconstruction, and it is the most widely accepted treatment modality [3].

Other common uses for vaginal stents include post-surgical support following surgeries such as vaginal reconstruction or the excision of vaginal tumors; a vaginal stent may be utilized to preserve the vaginal walls during their healing process. Regarding treatment for vaginal agenesis, for women who are born without a vagina, a vaginal stent may be a component of a plan to develop or preserve a vaginal canal [4]. In pelvic organ prolapse in cases where organs including the bladder, uterus, or rectum intrude into the vaginal region, a vaginal stent may be used temporarily to preserve the vaginal walls [5]. For patients who underwent radiation therapy for gynecological cancers, vaginal stents may be used to help maintain vaginal shape and prevent vaginal shortening or narrowing due to scarring [6]. It can also be employed in patients with gender dysphoria, who experience severe discomfort or difficulty due to the incongruence between their gender identity and their assigned gender at birth [7].

Materials used in the fabrication of vaginal stents include silicone and acrylic resin. However, their use is not without drawbacks, and clinicians must be aware of potential issues to optimize patient care and outcomes [8]. It is crucial to counsel the patient about post-insertion care needed to be taken to get the full benefits of a vaginal stent. But in some cases, post-insertion problems may occur, which need to be managed. The problems may be related to the material used, soft tissue injury caused by the stent, allergic reactions, etc., which will ultimately cause discomfort to the patient. Hence, we should manage them judiciously for better treatment outcomes.

The use of vaginal stents in reconstructive surgery has become a critical component in achieving excellent postoperative outcomes. These medical devices are key in preserving vaginal patency, promoting tissue healing, and reducing adhesions after surgical procedures. Despite their widespread usage and substantial breakthroughs in design and materials, vaginal stents do not come without risks. Troubleshooting vaginal stent difficulties is critical for healthcare providers to guarantee patient comfort, avoid complications, and improve the success rate of surgical procedures [9].

This review aims to provide a detailed summary of the most prevalent complications related to vaginal stents, including mechanical challenges, patient pain, and infection. It endeavors to provide clinicians with the knowledge they need to successfully manage and reduce these issues by investigating their origin and proposing evidence-based solutions. Healthcare workers can enhance patient outcomes and satisfaction by thoroughly understanding troubleshooting procedures, ultimately leading to advancements in postoperative care.

## Review

Potential issues

Hardness of the Stent

Discomfort and pain are two of the most immediate and serious consequences of vaginal stent hardness. The vaginal canal is a delicate location, and a firm stent can cause ongoing irritation. This discomfort might be slight or severe, greatly impairing the patient's quality of life. Pain can be more severe during movements like walking or sitting because the hard material can press against the vaginal walls and surrounding tissues. Specifically, the problem arises with the use of vaginal stents fabricated using acrylic. The acrylic vaginal stent, because of its hardness, causes discomfort to the patient, and hence patients find it difficult to perform normal daily activities because of hindrance caused by the hard and bulky acrylic made stent. Also, hard vaginal stents might injure the vaginal canal's delicate tissues [10].

The rigidity of the stent might cause friction with the mucosal lining, resulting in abrasions, ulcerations, or even erosions. Over time, recurrent trauma can cause severe tissue damage, raising the risk of infection and complicating the healing process. This problem can be overcome by using a different material for its fabrication, such as silicone, whose properties are superior to those of acrylic as it is soft and lightweight as compared to the acrylic stent [11]. Another method to overcome this problem is by fabricating a hollow vaginal stent which will reduce the weight of the stent thereby making it less bulky. The hollow vaginal stent can be fabricated by using ice, soap, salt, etc. during its fabrication [12].

Pain

Pain during daily activities might result after post-stent implantation, particularly when an acrylic stent is used. Strategies for reducing discomfort during dilation include the use of lidocaine jelly, estrogen cream, or oral analgesics. If the patient has pain while inserting the stent, increasing the quantity of lubricant may also be helpful [13]. Transitioning to a softer dilator or stent can help reduce pain in women with MRKH syndrome, as such patients may have reduced vaginal blood flow compared to normal women post-surgery [14].

Bleeding

Postoperative bleeding following surgery and after the placement of a stent is common. Examining the patient to check for trauma can be beneficial if they are bleeding. Mechanical trauma is one of the most common causes of vaginal stent-related bleeding. The insertion, presence, and removal of the stent may cause physical irritation and harm to the vaginal mucosa. Hard stents, in particular, can create abrasions, lacerations, or deeper injuries in the delicate vaginal tissues, resulting in bleeding. In case the stent causes excessive bleeding, it may need to be relocated, changed, or removed. To reduce damage and bleeding, alternative treatments or more flexible, biocompatible stent materials should be explored. Trauma may be avoided by utilizing a larger stent and increasing lubrication while insertion of the stent [15].

Fungal Infection

Often caused by Candida species. These infections can occur due to an imbalance between the vaginal microbiota and yeast. Mostly, the silicone vaginal stent is more prone to fungal infections [16]. To overcome this, silicone stents can be coated with polyethylene glycol (PEG) or propylene glycol. PEG is a stable, hydrophilic substance exhibiting no skin irritation and hence, as a surface coating, will enhance biocompatibility and promote tissue integration [17]. Additionally, research is being conducted on resorbable stents, which are made of shape memory foam based on polycaprolactone. These stents lower the risk of fungal infection and discomfort by providing enough radial force to maintain vaginal diameter during a four-week target healing time [16].

Malodor

Infection is a common cause of malodor associated with vaginal stents. The installation and continued presence of a stent can upset the natural balance of the vaginal microbiota, leaving it more vulnerable to bacterial or fungal infections. Common pathogens, such as Gardnerella vaginalis, which is linked to bacterial vaginosis, and Candida species, which cause yeast infections, can create foul-smelling discharge [18]. Infections can occur during stent insertion or as a result of the stent's interaction with normal vaginal flora. Vaginal stents can restrict the natural flow of vaginal fluids, resulting in buildup and stagnation. This retention can create an environment that encourages bacterial growth and the creation of unpleasant chemicals. The stent's presence can obstruct the vagina's normal cleansing systems, causing secretions to become trapped and acquire an unpleasant odor. 

If malodor is a result of infection or tissue necrosis, it may indicate a more serious underlying issue that requires prompt medical attention. Untreated infections can lead to complications such as pelvic inflammatory disease or other systemic infections [19]. The problem needs to be addressed as soon as detected as it can cause further infection due to bacterial overgrowth. This can be avoided by providing the patient with appropriate postoperative instructions, such as cleaning the stent with the use of a solution of vinegar or 25% povidone-iodine in water initially, and then an antibacterial soap solution can be used for cleaning followed by patting it dry with a clean paper towel to avoid an unpleasant stench [20]. If an infection is detected, appropriate antimicrobial therapy should begin. This could include antibiotics for bacterial infections or antifungal medicines for yeast infections. Maintaining strict hygiene measures during stent insertion and scheduling regular follow-ups can help prevent the problem.

Vaginal Stenosis

Scar tissue inevitably occurs following vaginal reconstruction when the canal lining heals over time, causing the vagina to become shorter and narrower. This condition is known as vaginal stenosis. Scar tissue undergoes a process known as scar contracture or scar maturation during healing because it lacks the elastic qualities of native, unmodified tissues. Stents are necessary following vaginoplasty to prevent vaginal stenosis because of the characteristics of wound healing and scar contracture [21]. Also, vaginal stents, especially those composed of hard or inflexible materials, can cause mechanical damage to the vaginal mucosa. This trauma can cause microtears, abrasions, or more serious injuries. As the body heals from these injuries, scar tissue might form, potentially leading to stenosis. Scar tissue is less flexible than normal vaginal tissue, and its growth can gradually narrow the vaginal canal.

Additionally, the presence of a stent can trigger a foreign body reaction [22]. The body’s immune system may perceive the stent as a foreign object and mount a response to isolate and encapsulate it. This reaction can involve the formation of granulation tissue and fibrosis, both of which contribute to the narrowing of the vaginal canal. As this is a patient-driven treatment, vaginal stenosis may still occur even after a vaginal stent has been placed due to the patient's negligence. It may also occur when a patient has not been kept on regular recall because, in certain situations, a different-sized vaginal stent may need to be made to maintain the stent's patency. Therefore, before being released from the ward, patients must demonstrate how to use the stent correctly. Hence, precise and easy-to-follow instructions for using the stent are essential to ensure the treatment's efficacy [23].

Deterioration and Discoloration

The materials used in vaginal stents, such as s polymers, can degrade over time due to exposure to bodily fluids, temperature changes, and mechanical stress, leading to weakening and eventual failure of the stent. Also, contact with bodily fluids and tissues can cause chemical reactions with the stent material, leading to degradation. For example, pH changes or enzymatic activity in the vaginal environment can contribute to material breakdown [24]. Moreover, continuous use of the stent can subject it to mechanical stresses such as bending, stretching, and compression, leading to fatigue and eventual failure of the material. Though none of the material is ideal in this case, eventually with time, both silicone and polymers tend to get discolored and deteriorate. To avoid these issues, patients should be counseled to avoid excessive bending, stretching, or manipulation that can stress the stent material [25]. Proper insertion and removal techniques should be explained to the patient to minimize damage to the stent. Approved lubricants or gels such as Surgilube or KY Jelly should be prescribed to the patient for lubrication during insertion or removal.

Allergic Reaction

While allergic reactions to acrylic vaginal stents are rare, they can occur. Allergic responses may manifest as local irritation, redness, itching, or discomfort. Allergy testing should be recommended to identify the specific allergen causing the reaction. In some cases, alternative materials (such as silicone-coated acrylic) may be considered to minimize the risk of allergies. Additionally, PACIENA prosthesis®, which is fitted to the typical vagina, can be utilized in these situations. It is made up of PLA (polylactic acid), a biocompatible substance with numerous biomedical applications; its effect on tissue growth has been shown in a variety of settings [26]. Also using stents with bioactive coatings, e.g., PEG can help in integration and healing. Close monitoring and prompt management in situations of aberrant tissue reactions are also essential for achieving the best possible results [27].

Vaginal Dryness

Vaginal dryness can occur post-surgery, and hence increasing the amount of lubricant may help if the patient has trouble inserting the dilator due to vaginal dryness; otherwise, it can cause severe pain and discomfort to the patient. In general, use of lubrication is necessary as the vaginal canal is lined with epithelium. Epithelial cells die as they make their way to the surface and slough off (exfoliation) [28]. The neovaginal lining sheds dead epithelial cells; hence, water-based lubricants, not silicone-based lubricants, are recommended. Silicone mixes with dead epithelial cells, which causes an unpleasant discharge. Silicone lubricants can also damage the medical grade silicone stents [29].

Patients' Discomfort and Compliance

It is important to consider the patient's mental health throughout the entire treatment, as those who have vaginal agenesis or dysfunction also tend to have greater rates of depression and body image difficulties, as well as sexual dysfunction. These factors often result in poor patient compliance with stent therapy, leading to suboptimal treatment outcomes [30]. As part of continuing care, these issues must be addressed. It is best to encourage them to seek counseling, preferably from an experienced therapist. Hence, patient education and involvement in the decision-making process for stent selection are critical. Using softer, more flexible stents and ensuring a correct fit can improve patient comfort. Furthermore, giving proper pain treatment and assistance during the recovery phase can boost compliance [31].

Discussion

Post-surgery, vaginal stents are used to preserve the depth, width, length, and patency of the newly formed neovaginal structure and to avoid its contraction or shrinkage. They also function as a hemostatic agent. According to the results of a comprehensive review, stents or dilators are advantageous and improve the well-being of women with a history of stenosis. Veyssi et al. stated that several different materials for vaginal stent fabrication are evolving; for years, sterile gauze, candle wax, vulcanized rubber, Styrofoam, and silicone foam have been used; with technical advancements in materials, nowadays, silicone fabricated stent has gained popularity. Also, vaginal stents with surface coatings enhance biocompatibility and promote tissue integration [23]. Coskon et al. (2007) recommended the use of silicone material vaginal stent fabrication due to its excellent material properties as well as its lightweight. However, the major problem with silicone material is bacterial adhesion, and hence it is not the most acceptable material for fabrication of the stent [8].

Every material has some drawbacks that need to be addressed by taking into consideration the physical, mental, and emotional well-being of the patient. Vaginal stents should be designed for maximum, long-term therapeutic results rather than temporary, anatomical fit. The stents need to be devised with non-porous material that is easy to clean after each use. Zhao et al. stated that rigid stents can cause contracture formation and graft loss or fibrosis and also pressure-related bladder or rectum perforations [32]. The frequency of these complications has dropped with the use of soft stents. It should be fabricated by taking into consideration the exact vaginal length and diameter with a slightly curved and tapered tip that is helpful for easy insertion and removal. The stents should improve graft take, should be simple to replicate, practical, economical, and time-efficient. It should be biocompatible, causing no harm to the tissue as the vagina is lined by stratified squamous, non-keratinizing epithelium lining that is thin and distensible.

Hicks et al. (2024) in their study discussed self-fitting vaginal stents based on shape-memory polymers (SMPs). They mentioned that with the use of SMPs, material properties such as transition temperature, Young's modulus, and maximum elongation can be more precisely tailored to satisfy functional engineering requirements. Along with restoring the normal anatomy, the psychological conditions of the patient should be taken into utmost consideration [33]. The stent mainly serves the purpose of maintaining the surgically created neovagina after vaginal reconstruction by preventing fibrosis, but while doing so, it should not cause any discomfort to the patient and should improve the patient's overall quality of life.

Before surgery, all patients should be advised that dilator therapy should be started as soon as possible to prevent stenosis. Also, before surgery, patients must be evaluated for dilation readiness. Though a lot of research has been done on intraoperative procedures, not much has been written about the best time or duration for postoperative stenting. Vaginal stents are usually left in place postoperatively for vaginoplasty treatments that necessitate grafts or significant mobilization of the native vagina. Patients are advised to either wear a stent continuously for three to six months after being discharged or to wear a stent while sleeping at first. After surgery, careful monitoring is required. Patients should be encouraged to return as soon as possible (after five days of the surgery, and then after regular intervals of two, four, and eight weeks [34].

By implementing effective maintenance strategies, optimizing stent design, and prioritizing patient-centered care, clinicians can enhance the functionality of vaginal stents and improve overall treatment success rates. The successful management of vaginal stent-related problems necessitates a comprehensive approach that incorporates technology improvements, patient-centered care, and ongoing education for healthcare personnel. By addressing the mechanical, biological, and socioeconomic elements of stent use, we can improve patient outcomes and increase the overall effectiveness of vaginal stents.

## Conclusions

Vaginal stents are associated with several difficulties and adverse effects that need to be carefully managed and monitored, despite their therapeutic advantages. For optimal treatment outcomes and patient satisfaction, clinicians should evaluate the risks and benefits of stent therapy on a case-by-case basis and be alert when addressing patient concerns and adverse events. Future vaginal stent designs will need to be safer and more tolerable, which will require further research and technological developments. Continued innovation, interdisciplinary collaboration, and patient-centered approaches are crucial for harnessing the full potential of advanced vaginal stents in improving women's health outcomes and quality of life.

## References

[1] Beri A, Pisulkar S, Bansod A, Shrivastava A (2023). Case report: digitally fabricated acrylic vaginal stent for a female with isolated vaginal agenesis. F1000Res.

[2] Romanski PA, Bortoletto P, Pfeifer SM (2021). Creation of a novel inflatable vaginal stent for McIndoe vaginoplasty. Fertil Steril.

[3] Panici PB, Ruscito I, Gasparri ML, Maffucci D, Marchese C, Bellati F (2011). Vaginal reconstruction with the Abbè-McIndoe technique: from dermal grafts to autologous in vitro cultured vaginal tissue transplant. Semin Reprod Med.

[4] Lyons ME, Goldman JJ (2023). Vulvar-Vaginal Reconstruction. StatPearls.

[5] Aboseif C, Liu P (2022). Pelvic Organ Prolapse. https://www.ncbi.nlm.nih.gov/books/NBK563229/.

[6] Johnson N, Miles TP, Cornes P (2010). Dilating the vagina to prevent damage from radiotherapy: systematic review of the literature. BJOG.

[7] Kamalakannan J, Murthy V, Kularashmi BS, Jajoo K (2015). Customized silicone vaginal stent. J Obstet Gynaecol India.

[8] Coskun A, Coban YK, Vardar MA, Dalay AC (2007). The use of a silicone-coated acrylic vaginal stent in McIndoe vaginoplasty and review of the literature concerning silicone-based vaginal stents: a case report. BMC Surg.

[9] Buss JG, Lee RA (1989). McIndoe procedure for vaginal agenesis: results and complications. Mayo Clin Proc.

[10] Chung RK, Findley J, Flyckt RL (2021). The mother of invention-introduction of a novel inflatable stent for McIndoe neovagina. Fertil Steril.

[11] Rathee M, Chauhan M, Jain P, Shetye A (2020). Customized hollow surgical stent for congenital vaginal agenesis in early adolescent female with MRKH syndrome: a case report. Niger J Plast Surg.

[12] Amies Oelschlager AM, Debiec K (2019). Vaginal dilator therapy: a guide for providers for assessing readiness and supporting patients through the process successfully. J Pediatr Adolesc Gynecol.

[13] Damast S, Jeffery DD, Son CH, Hasan Y, Carter J, Lindau ST, Jhingran A (2019). Literature review of vaginal stenosis and dilator use in radiation oncology. Pract Radiat Oncol.

[14] Raza FB, Raju K, Babu A, Vaidyanathan AK, Mehta BS (2023). Customised vaginal stent-the phases in management of vaginal agenesis in Mayer-Rokitansky-Küster-Hauser (MRKH) syndrome. J Family Reprod Health.

[15] Wancura M, McCracken JM, Steen E, Cosgriff-Hernandez E, Keswani S, Hakim JC (2019). Emerging technologies in pediatric gynecology: new paradigms in women's health care. Curr Opin Obstet Gynecol.

[16] Liu M, Juravic M, Mazza G, Krychman ML (2021). Vaginal dilators: issues and answers. Sex Med Rev.

[17] Kwun Kim S, Hoon Park J, Cheol Lee K, Min Park J, Tae Kim J, Chan Kim M (2003). Long-term results in patients after rectosigmoid vaginoplasty. Plast Reconstr Surg.

[18] Haddad NC, Soares Brollo LC, Pinho Oliveira MA, Bernardo-Filho M (2021). Diagnostic methods for vaginal stenosis and compliance to vaginal dilator use: a systematic review. J Sex Med.

[19] Mogilnicka I, Bogucki P, Ufnal M (2020). Microbiota and malodor - etiology and management. Int J Mol Sci.

[20] Alaniz V, Laurin J, Huguelet P, Scott S, Appiah L, Fasano M, Hampanda K (2022). Vaginal stents: what is working and what is needed to prevent vaginal stenosis in young women. J Pediatr Adolesc Gynecol.

[21] Krychman M, Liu MC, Daniel Meller BS (2020). Vaginal dilators: a guide for health care professionals. Contemp OB/GYN.

[22] Lim CK, Yang JB, Lee JY (2024). Three-dimensional printed mold for neovaginal cavity maintenance in vaginal agenesis: a case report. Med Case Rep Stud Protoc.

[23] Acién P, Nohales-Alfonso FJ, Sánchez-Ferrer ML, Sánchez-Lozano M, Navarro-Lillo V, Acién M (2019). Clinical pilot study to evaluate the neovaginal PACIENA prosthesis® for vaginoplasty without skin grafts in women with vaginal agenesis. BMC Womens Health.

[24] Stahl JM, Qian JM, Tien CJ (2019). Extended duration of dilator use beyond 1 year may reduce vaginal stenosis after intravaginal high-dose-rate brachytherapy. Support Care Cancer.

[25] Akbaba S, Oelmann-Avendano JT, Krug D (2019). The impact of vaginal dilator use on vaginal stenosis and sexual quality of life in women treated with adjuvant radiotherapy for endometrial cancer. Strahlenther Onkol.

[26] Cerentini TM, Schlöttgen J, Viana da Rosa P (2019). Clinical and psychological outcomes of the use of vaginal dilators after gynaecological brachytherapy: a randomized clinical trial. Adv Ther.

[27] Rathee M, Boora P, Kundu R (2014). Custom fabricated acrylic vaginal stent as an adjunct to surgical creation of neovagina for a young female with isolated vaginal agenesis. J Hum Reprod Sci.

[28] Veyssi A, Robinson A, Cosgriff Hernadez E (2023). Self-fitting vaginal stents from biodegradable, shape memory polymers. J Pediatr Adolesc Gynecol.

[29] Zhao M, Li P, Li S, Li Q (2009). Use of autologous micromucosa graft for vaginoplasty in vaginal agenesis. Ann Plast Surg.

[30] Morgan O, Lopez MD, Martinez AJ, Marshall DC, Schnur JB (2022). Systematic review of comparisons between plastic and silicone dilators: revealing a knowledge gap. Sex Med Rev.

[31] Hicks AJ, Roberts C, Robinson A (2024). Polycaprolactone-based shape memory foams as self-fitting vaginal stents. bioRxiv.

[32] Osmałek T, Froelich A, Jadach B (2021). Recent advances in polymer-based vaginal drug delivery systems. Pharmaceutics.

[33] Anderson DJ, Marathe J, Pudney J (2014). The structure of the human vaginal stratum corneum and its role in immune defense. Am J Reprod Immunol.

[34] Lee Y (2018). Patients' perception and adherence to vaginal dilator therapy: a systematic review and synthesis employing symbolic interactionism. Patient Prefer Adherence.

